# Supplementary material for: Imidazo-Pyrazole-Loaded Palmitic Acid and Polystyrene-Based Nanoparticles: Synthesis, Characterization and Antiproliferative Activity on Chemo-Resistant Human Neuroblastoma Cells
Source: Int J Mol Sci. 2023 Oct 9;24(19):15027. doi: 10.3390/ijms241915027 (PMC10573130; doi:10.3390/ijms241915027)
Supplement: Supplementary file 1 [file ijms-24-15027-s001.zip › ijms-2628739-supplementary.pdf]

Supplementary Materials

Imidazo-pyrazole-Loaded Palmitic Acid and Polystyrene-Based Nanoparticles: Synthesis, Characterization and Antiproliferative Activity on Chemoresistant Human Neuroblastoma Cells

Giulia Elda Valenti [1], Barbara Marengo [1], Marco Milanese [2], Guendalina Zuccari [3], Chiara Brullo [3], Cinzia Domenicotti [1,*], and Silvana Alfei [2,*]

[1] Department of Experimental Medicine (DIMES), University of Genova, Via Alberti L.B., 16132 Genoa, Italy; giuliaelda.valenti@edu.unige.it (G.E.V.); barbara.marengo@unige.it (B.M.)

[2] Department of Pharmacy, Section of Chemistry and Pharmaceutical and Food Technologies, University of Genoa, Viale Cembrano, 4, 16148 Genoa, Italy

[3] Department of Pharmacy (DIFAR), Section of Medicinal Chemistry and Cosmetic Product, University of Genoa, Viale Benedetto XV, 3, 16132 Genoa, Italy; chiara.brullo@unige.it (C.B.); guendalina.zuccari@unige.it (G.Z.); marco.milanese@unige.it (M.M.)

* Correspondence: cinzia.domenicotti@unige.it (C.D.); alfei@difar.unige.it (S.A.); Tel.: +39-010-355-2296 (S.A.)

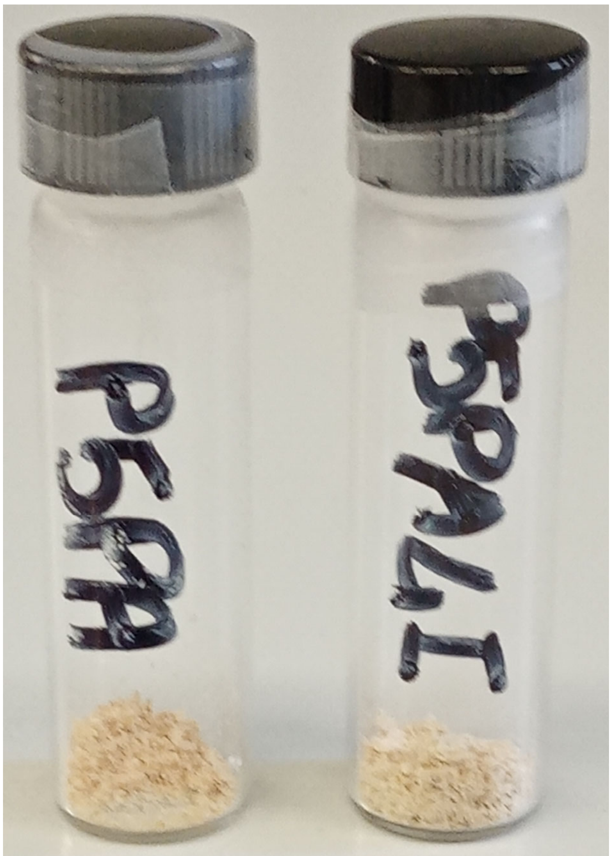

Figure S1. Appearance of P5PA and P%PA-4I NPs.

Video S1: https://clipchamp.com/watch/O9s7mm5bxkS.

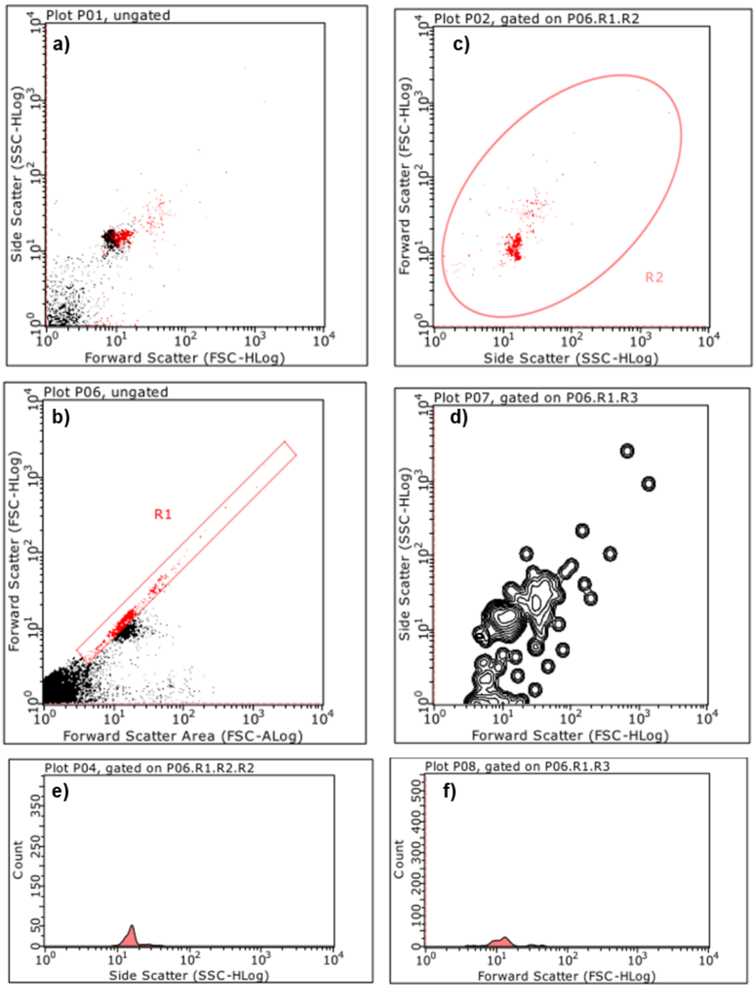

Figure S2. Type I deionized water 0.22µM filtered. Representative flow cytometry graphs for: ungated dot plot Forward Scatter high (FSC-HLog) vs. Side Scatter high (SSC-HLog), R1 gated particles in red (**a**); ungated dot plot Forward Scatter Area (FSC-ALog) vs. Forward Scatter High (FSC-HLog), R1 gated particles in red (**b**); R2 gated dot plot Side Scatter High (SSC-HLog) vs. Forward Scatter High (FSC-HLog) (**c**); R3 gated contour plot Side Scatter High (SSC-HLog) vs. Forward Scatter High (FSC-HLog) (**d**). Representative flow cytometry histogram plots showing the Side Scatter High (SSC-HLog) and the Forward Scatter High (FSC-HLog) distributions. 33602 events per group were acquired (**e**,**f**).

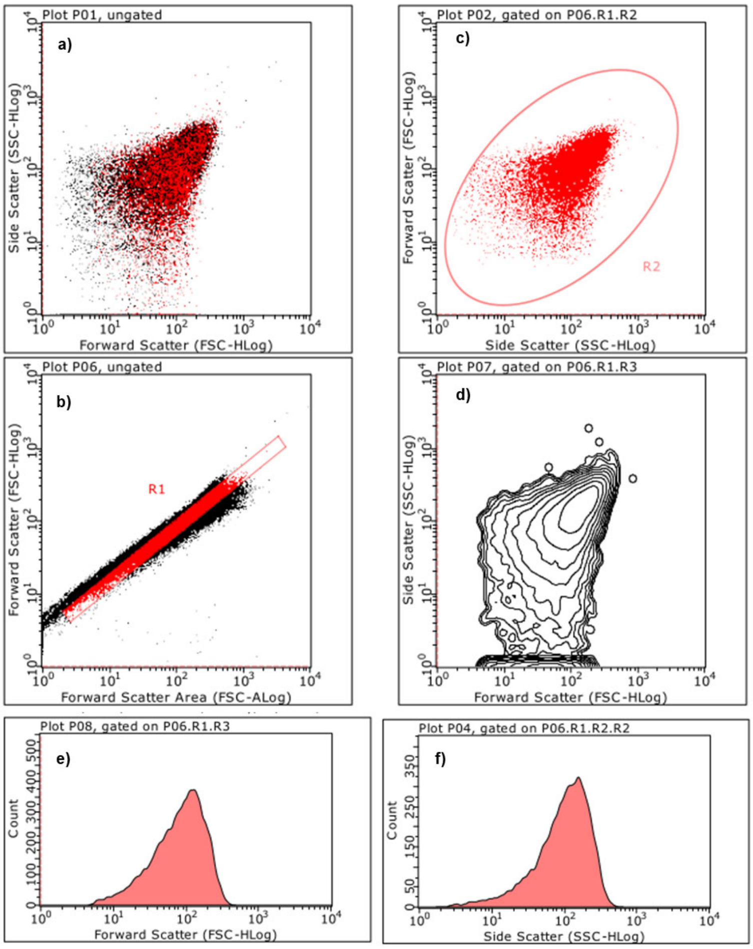

Figure S3. Unstained precision size polystyrene standard beads 1µm diameter (Polysciences Europe GmbH, Hirschberg an der Bergstrasse, Germany, cat# 64030-15). Representative flow cytometry graphs for: ungated dot plot Forward Scatter high (FSC-HLog) vs. Side Scatter high (SSC-HLog), R1 gated particles in red (**a**); ungated dot plot Forward Scatter Area (FSC-ALog) vs. Forward Scatter High (FSC-HLog), R1 gated particles in red (**b**); R2 gated dot plot Side Scatter High (SSC-HLog) vs. Forward Scatter High (FSC-HLog) (**c**); R3 gated contour plot Side Scatter High (SSC-HLog) vs. Forward Scatter High (FSC-HLog) (**d**). Representative flow cytometry histogram plots showing the Forward Scatter High (FSC-HLog) and the Side Scatter High (SSC-HLog) distributions. 33602 events per group were acquired (**e**,**f**).

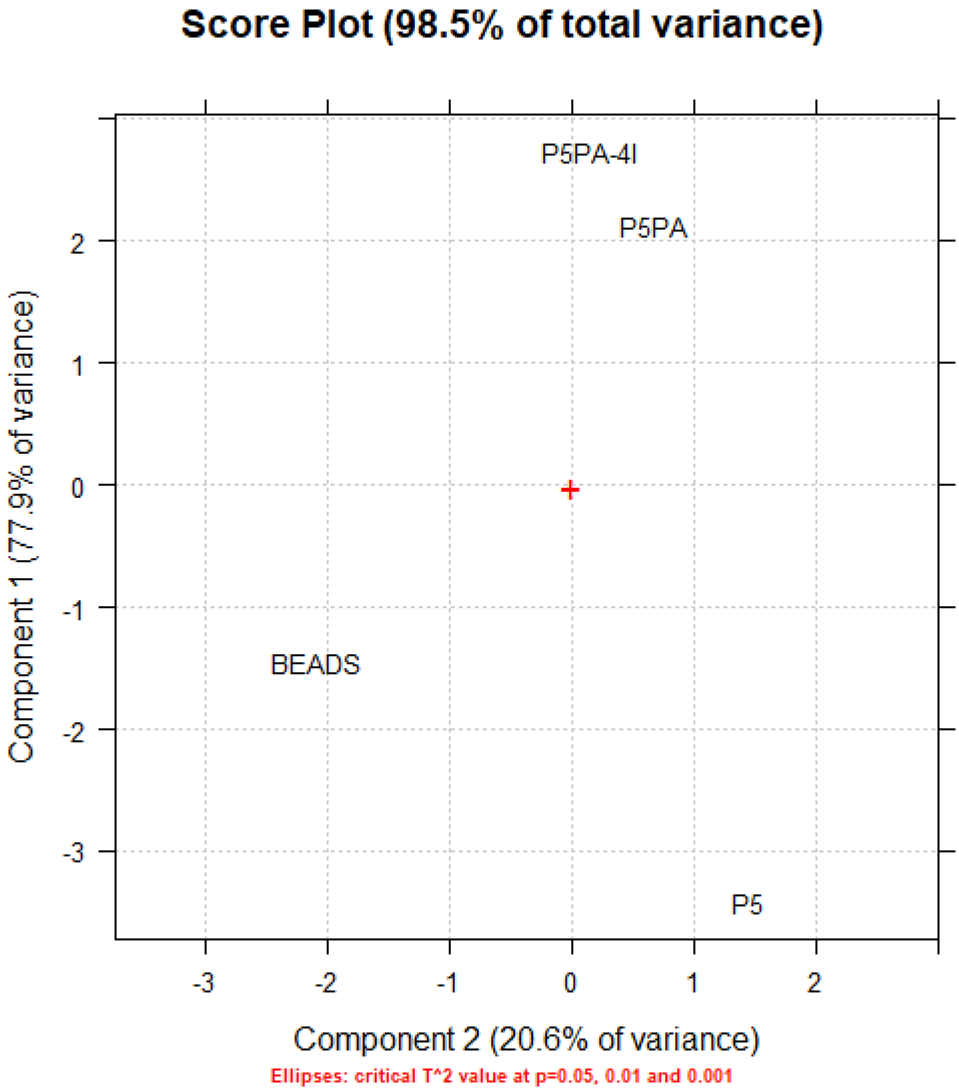

Figure S4. Score plot of PC2 (explaining the 20.6% of variance) vs. PC1 (explaining for the 77.9% of variance) of obtained by PCA on statistic data by cytofluorimetric analyses on polystyrene standard beads (1 µm), P5, P5PA and P5PA-4I.

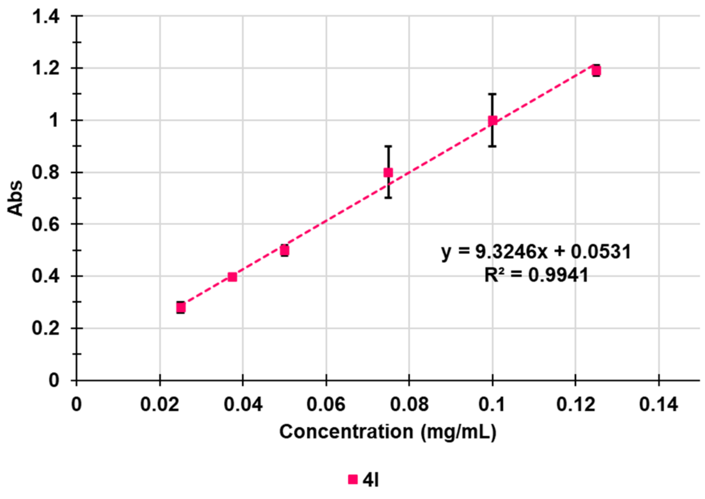

Figure S5. Linear calibration model of **4I**.

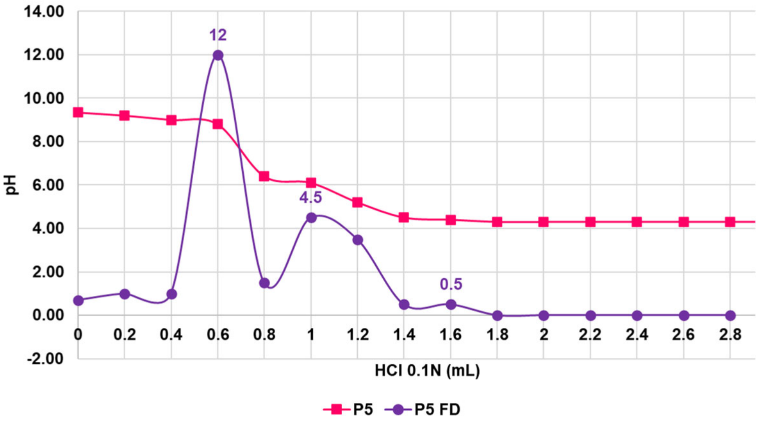

Figure S6. Titration curve of P5 (fuchsia line with square indicators) and related first derivative (FD), purple line with round indicators).

Section S1.

Even if we know that it could be a mere speculation, we tried to estimate the milliequivalents of **4I** present in 1 gram of P5PA-4I by means of the volume of HCl 0.1 N consumed between the third and the fourth end point (0.5 mL) and the result was compared to that obtained by UV-Vis analyses (Figure 17b in the main text, blue line). Briefly, we first estimated the equivalents of basic nitrogen atoms of **4I** (0.822 mmol/g) and knowing that basic nitrogen atoms for equivalent of **4I** are two, we obtained those of **4I** making 0.822/2 (0.411 mmol/g). According to the MW of **4I**, it resulted that **4I** in P5PA-4I was 192.1 mg/g, while in the prepared 402.0 mg, **4I** was 77.2 mg, corresponding to a slightly underestimated DL% of 19.2% vs the 20.9% estimated by UV-Vis spectroscopy. Similarly, we tried to estimate only the primary amine groups of P5 in P5PA-4I using the HCl 0.1N volume between the second and third end point (3 mL). In this regard, the milliequivalents of NH_2 of P5 per gram of P5PA-4I NPs were 4.9 mmol/g. So, considering that, according to Table 2 (main text), 1-gram P5PA-4I contains 0.517 g of P5, the milliequivalents of primary amine groups per gram of P5 estimated here were 9.5 mmol/g vs. 12.5 mmol/g estimated titrating P5 alone. These finding evidenced that, while for the simple nanocomposites as P5PA, titration can be a reliable analytical method to estimate their composition, for more complex nanocomposites as P5PA-4I containing differently basic nitrogen atoms titration is not.

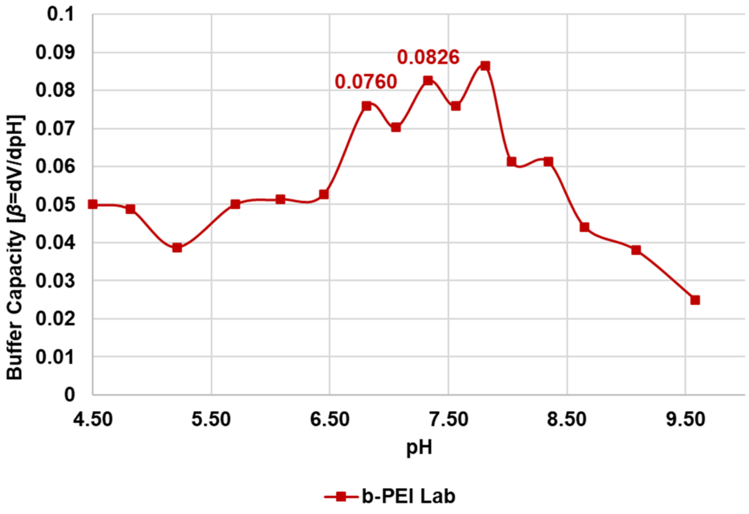

Figure S7. Buffer capacity of branched PEI 25K.

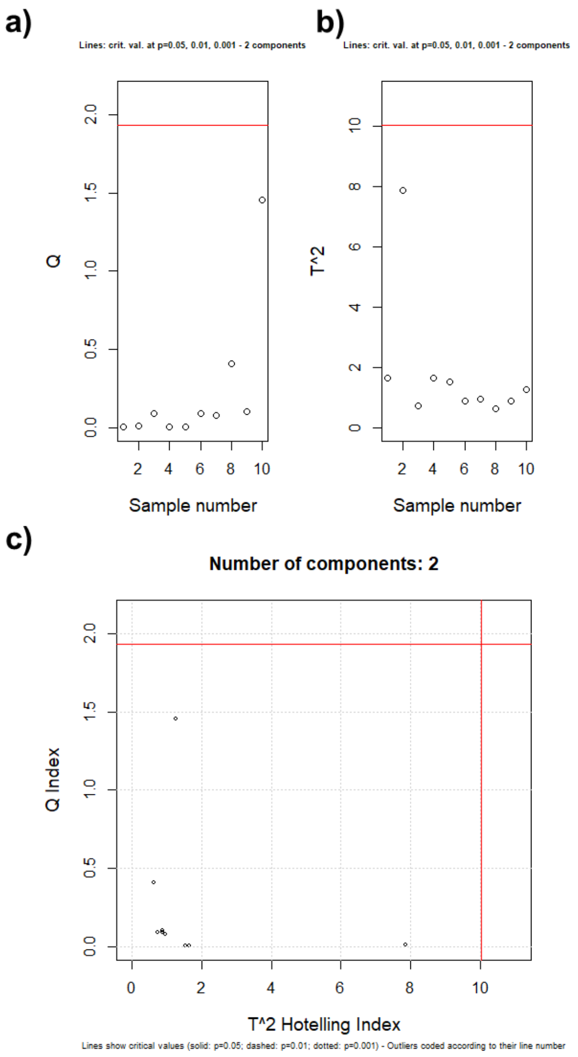

Figure S8. Q (**a**) and T^2 (**b**) contribution plots and the influencing plot of T^2 Hotelling index vs Q index (**c**). The red solid lines indicate the more restrictive critical values ($p = 0.05$). Other lines ($p = 0.01$ and $p = 0.001$ have been not shown).

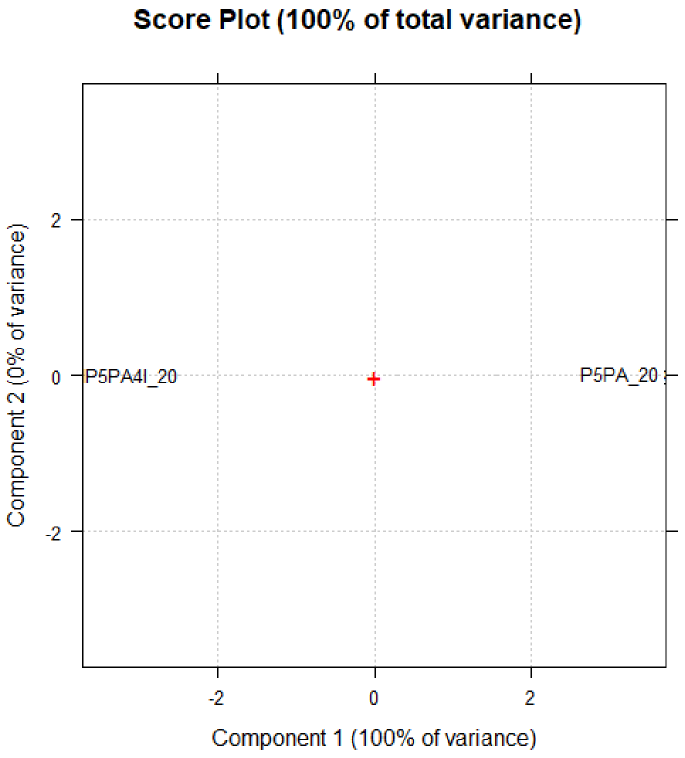

Figure S9. Score plot of PC1 (explaining the 100% of variance) vs. PC2 (explaining for the 0% of variance) of P5PA_20 and P5PA4I_20.

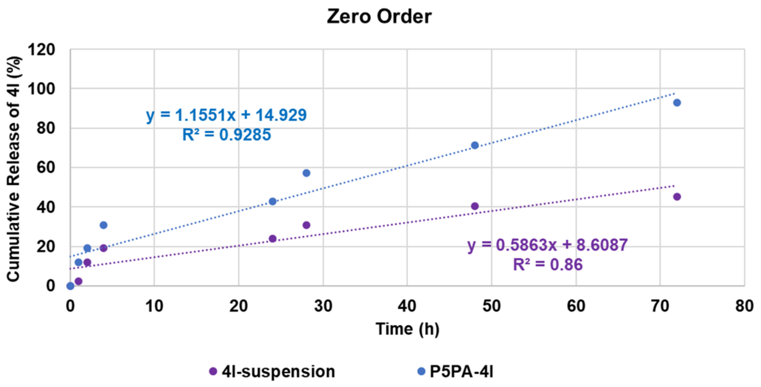

Figure S10. Zero-order kinetic models.

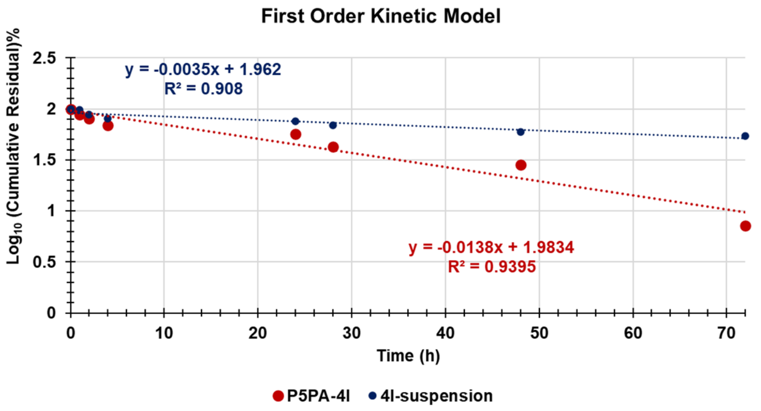

Figure S11. First order kinetic models.

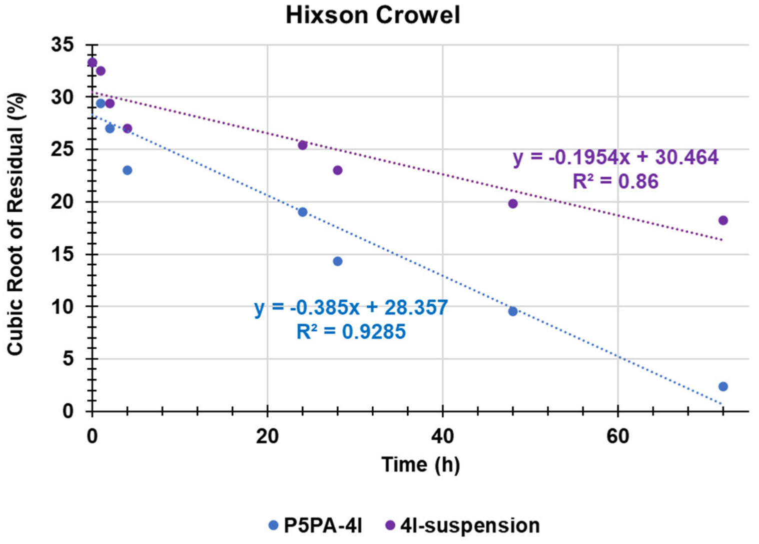

Figure S12. Hixson Crowel kinetic models.

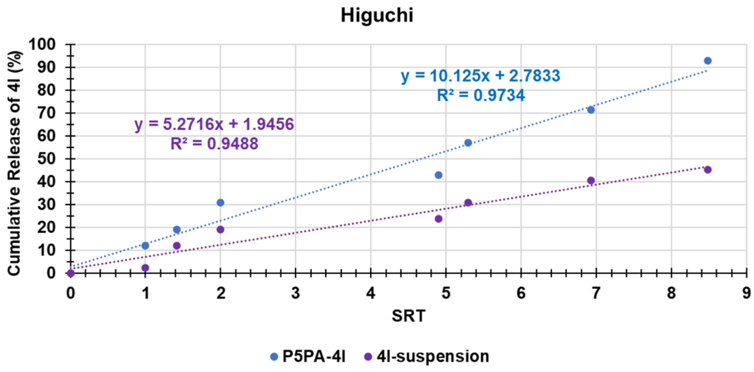

Figure S13. Higuchi kinetic models. SRT = square root of times.

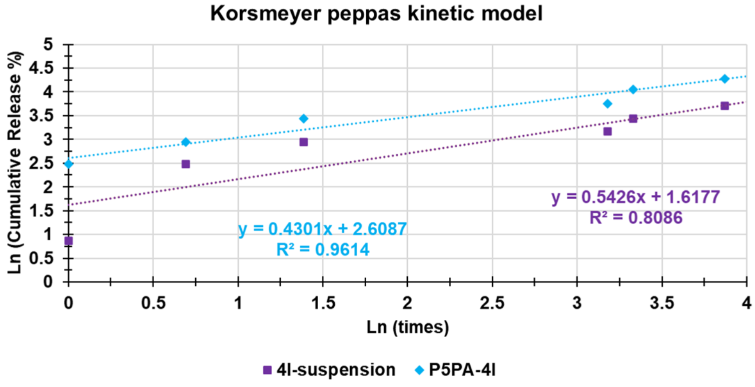

Figure S14. Korsmeyer peppas kinetic models.

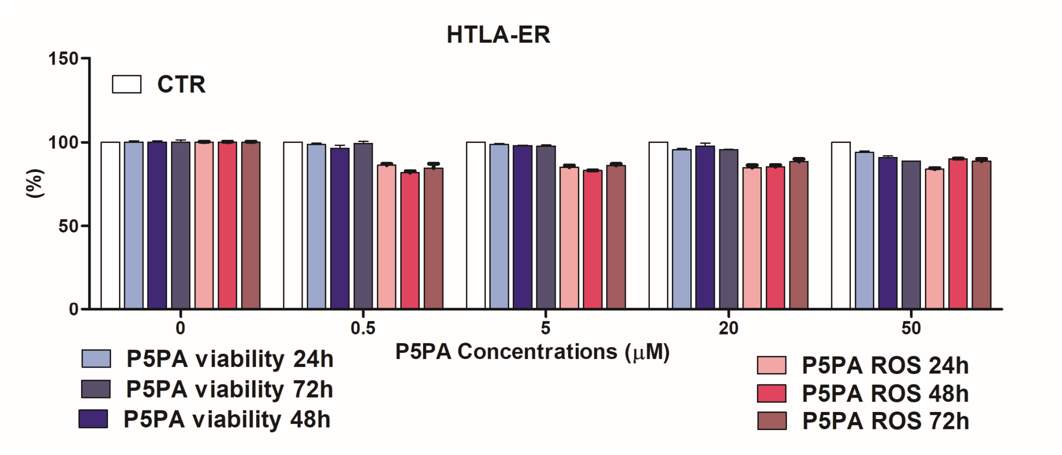

Figure S15. Dose- and time-dependent biological effects of P5PA NPs in HTLA-ER cells.

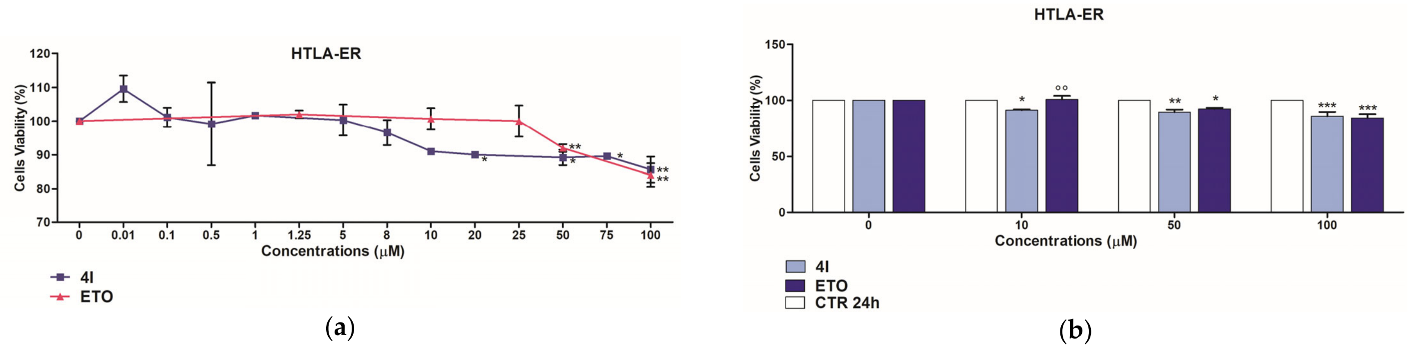

Figure S16. Dose- dependent cytotoxic effects of **4I** and ETO in HTLA-ER cells at 24 hours of exposure at all concentrations tested for both compounds (a), and only when administered at the same dose for direct comparison (b). Significance refers to control (*) or **4I** (°). Specifically, $p > 0.05$ ns; $p < 0.05$ * (vs. CTR); $p < 0.01$ ** (vs. CTR), °° (vs. **4I**); $p < 0.001$ *** (vs. CTR).

Disclaimer/Publisher's Note: The statements, opinions and data contained in all publications are solely those of the individual author(s) and contributor(s) and not of MDPI and/or the editor(s). MDPI and/or the editor(s) disclaim responsibility for any injury to people or property resulting from any ideas, methods, instructions or products referred to in the content.
